# Supplementary material for: The Prevalence, Risk Factors, and Outcomes of Hepatitis E Virus Infection in Solid Organ Transplant Recipients in a Highly Endemic Area of Italy
Source: Viruses. 2025 Mar 31;17(4):502. doi: 10.3390/v17040502 (PMC12031106; doi:10.3390/v17040502)
Supplement: Supplementary file 1 [file viruses-17-00502-s001.zip › viruses-3531041-supplementary.pdf]

Supplementary Table S1. Distribution of the different HEV markers in SOT recipients.

Recipient IN	Anti-IgM OD	Anti-IgM qr	Anti-IgG OD	Anti-IgG qr	HEV RNA copies/ml
18	0.000	Negative	**0.311***	**Positive**	NR
25	0.000	Negative	**0.923**	**Positive**	NR
34	0.001	Negative	**2.101**	**Positive**	NR
36	**1.202**	**Positive**	**1.940**	**Positive**	NR
39	0.001	Negative	**1.600**	**Positive**	NR
43	0.000	Negative	**0.541**	**Positive**	NR
44	0.001	Negative	**2.516**	**Positive**	NR
45	0.000	Negative	**0.950**	**Positive**	NR
46	0.001	Negative	**0.471**	**Positive**	NR
52	0.039	Negative	**1.404**	**Positive**	NR
54	0.029	Negative	**2.530**	**Positive**	NR
55	0.000	Negative	**0.275**	**Positive**	NR
57	0.000	Negative	**2.458**	**Positive**	NR
58	0.001	Negative	0.008	**Negative**	**10^4-10^5**
59	0.002	Negative	**1.768**	**Positive**	NR
72	0.031	Negative	**1.818**	**Positive**	NR
73	0.027	Negative	**2.455**	**Positive**	NR
74	0.001	Negative	**0.713**	**Positive**	NR
82	0.103	Negative	**2.497**	**Positive**	NR
84	**2.312**	**Positive**	**3.000**	**Positive**	**$>10^5$**
87	0.002	Negative	**0.322**	**Positive**	NR
92	**0.523**	**Positive**	**2.479**	**Positive**	NR
97	0.173	Negative	**2.541**	**Positive**	NR
99	0.000	Negative	**2.493**	**Positive**	NR
107	0.000	Negative	**1.268**	**Positive**	NR
108	0.088	Negative	**0.607**	**Positive**	NR
119	**2.550**	**Positive**	**2.545**	**Positive**	**10^5**
126	0.000	Negative	**0.683**	**Positive**	NR
132	0.001	Negative	**0.679**	**Positive**	NR
133	**1.270**	**Positive**	0.001	**Negative**	**$< 10^2$**
138	0.001	Negative	**1.202**	**Positive**	NR
141	**0.538**	**Positive**	**1.526**	**Positive**	NR
145	0.001	Negative	**2.500**	**Positive**	NR
158	0.016	Negative	**1.763**	**Positive**	NR
160	0.001	Negative	**0.210**	**Positive**	NR
161	0.012	Negative	**0.671**	**Positive**	NR
166	0.000	Negative	**0.243**	**Positive**	NR
174	0.139	Negative	**2.493**	**Positive**	NR
175	0.001	Negative	**2.246**	**Positive**	NR
197	0.070	Negative	**0.285**	**Positive**	NR
199	0.000	Negative	**2.494**	**Positive**	NR
200	0.000	Negative	**2.533**	**Positive**	NR
204	0.000	Negative	**2.467**	**Positive**	NR
205	0.000	Negative	**1.088**	**Positive**	NR
213	0.003	Negative	**1.970**	**Positive**	NR
215	0.002	Negative	**1.266**	**Positive**	NR

218	**0.350**	**Positive**	**1.746**	**Positive**	NR
223	0.004	Negative	**0.378**	**Positive**	NR
226	0.001	Negative	**0.589**	**Positive**	NR
228	**2.535**	**Positive**	**1.190**	**Positive**	**10^3**
234	0.012	Negative	**1.279**	**Positive**	NR
236	0.000	Negative	**2.475**	**Positive**	NR
237	0.002	Negative	**2.532**	**Positive**	NR
242	0.001	Negative	**2.490**	**Positive**	NR
252	0.001	Negative	**2.448**	**Positive**	NR
262	0.001	Negative	**0.824**	**Positive**	NR
265	0.013	Negative	**0.638**	**Positive**	NR
267	0.001	Negative	**2.557**	**Positive**	NR
272	0.000	Negative	**2.491**	**Positive**	NR
275	0.000	Negative	**0.929**	**Positive**	NR
287	**0.834**	Negative	**2.361**	**Positive**	NR
292	0.001	Negative	**1.510**	**Positive**	NR
294	0.001	Negative	**0.958**	**Positive**	NR

IN, Identification Number; OD, Optical Density; qr, qualitative result; NR, non-reactive.

* Positive test results are highlighted in bold
